# Design and Experimental Study of a Large Beam Waist Streak Tube in an ICF Experiment

**DOI:** 10.3390/s23063158

**Published:** 2023-03-15

**Authors:** Hou-zhi Cai, Xuan Deng, Li-hong Niu, Qin-lao Yang, Jing-jin Zhang

**Affiliations:** 1 Key Laboratory of Optoelectronic Devices and Systems, Ministry of Education, Shenzhen 518060, China; 2 College of Physics and Optoelectronic Engineering, Shenzhen University, Shenzhen 518060, China

**Keywords:** X-ray, streak tube, multi-frame, in situ framing

## Abstract

In order to realize in situ multi-frame framing, this paper designed and developed a large-waist framing converter tube. The size ratio between the waist and the object was about 1.16:1. The subsequent test results showed that the static spatial resolution of the tube could reach 10 lp/mm (@ 72.5%) under the premise of this adjustment, and the transverse magnification could reach 2.9. Once the MCP (Micro Channel Plate) traveling wave gating unit is equipped at the output end, it is expected to promote the further development of in situ multi-frame framing technology.

## 1. Introduction

The photoelectric technology-based framing camera is an indispensable framing imaging device in the field of X-ray ultrafast diagnosis. Furthermore, it has important applications in the research of ultrafast phenomena, such as femtosecond lasers, plasma radiation, and nuclear fusion, because of its two-dimensional spatial resolution and picosecond temporal resolution. Especially in the research of the implosion process of inertial confinement fusion (ICF), it can intuitively describe the formation and evolution of the ICF implosion hot spot, thus providing an important judgement basis for whether the ICF experiment meets the ignition conditions [1,2,3,4,5,6,7,8,9,10,11,12,13,14]. Additionally, it can be an irreplaceable two-dimensional spatial visual detection tool.

Due to the extremely short duration of hot spots (about hundreds of picoseconds), it is required to provide two-dimensional spatial information in the diagnosis process and to have the ability of high temporal resolution. One of the main methods for observing hot spots is the pinhole array (or KB microscope) with the X-ray traveling wave gated framing imaging technology. However, due to the distances between pinholes in the pinhole array, which may correspond to the different azimuth of the measured target, the result is that the in-situ amplitude division cannot be realized.

The Livermore Laboratory of the United States once proposed a single line-of-sight (SLOS) framing technology [15,16,17]. This technology uses a mechanical cutting method to divide the electron beam, which can achieve strict SLOS framing. Furthermore, we conducted a follow-up study on this SLOS framing technology [18], which verified the effectiveness of this framing technology. Additionally, the technology can only achieve four framing images, limited by the size of the “cross” image analyzer and the size of the electron beam spot. In addition to upgrading the structure of the image dissector to 9 or 16 frames, it is crucial to increase the beam waist size of its electron beam to obtain more frames and avoid limiting its final number.

However, to improve the scanning deflection sensitivity and its technical temporal resolution, the waist radius of the electron beam should be as small as possible for the scanning streak tube with high spatial and temporal resolutions, which is contrary to the requirements of the multi-frame SLOS technology for the streak tube. In addition, the electron beam waist and the imaging quality of the electron optical imaging system are a pair of contradictions. A larger beam waist will increase the field curve, thus making it difficult to obtain a high-quality image.

In this study, a streak tube with a large beam waist radius was designed and developed to meet this requirement. The experimental results showed that the ratio of the waist-to-object size was 1.16:1, which was significantly better than the ratio of 0.72:1 of the streak tube used in the previous tracking research. Moreover, the results showed a static spatial resolution that could still reach at least 10 lp/mm (@ CTF 42%). Once the MCP (Micro Channel Plate) traveling wave gating unit is equipped at the output end, it is expected to promote the further development of SLOS technology.

The structure of this paper is as follows: Section 1 provides the introduction. Section 2 discusses the structural design concept of the streak tube. Section 3 describes the development of the streak sample tube, including the preparation of the photocathode, fluorescent screen, etc. Section 4 discusses the experimental test and result analysis, and Section 5 provides conclusions.

## 2. Design Idea

In the electron optical imaging system, there is a generally valid Lagrange–Helmholtz relation [19]:

(1) M×β=θ×εo×cos2θφtotal+εo×cos2θ

where M is the lateral magnification, $\varphi_{total}$ is the total anode pressure of the streak tube, $\varepsilon_{o}$ is the most probable energy of the photocathode material, θ is the initial emission angle of the electron beam, and β is the angle of the image side electron beam. For the same streak tube, the total anode pressure $\varphi_{total}$ and the photocathode materials are the same, but the initial emission angle θ conforms to the Lambert emission law. Therefore, for Equation (1), the right-hand side is equal to a constant.

**Figure 1:**
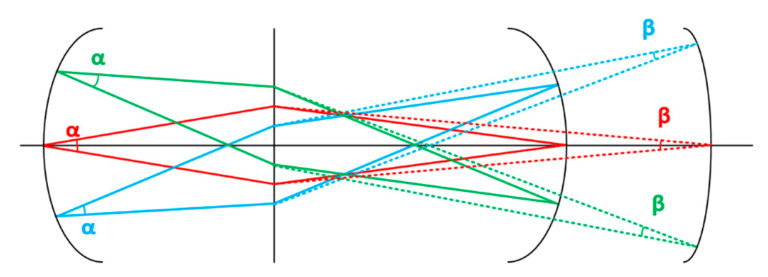
Relationship between the image filed angle and the field curvature: the solid line represents the electron track of the large image field angle and the Petzval image plane, while the dotted line corresponds to the small one (The red line represents the envelope diagram of the electron beam trajectory emitted by the object point on the axis, the green and blue represent the emission of the off-axis object point respectively, wherein the green represents the object point emitted on the positive half axis, and the blue represents the object point emitted on the negative half axis).

For the electron beam in the electron optical imaging system, the large beam waist refers to a larger image angle   β, which will lead to a smaller imaging depth of the field of the streak tube and further lead to a larger field curve. Therefore, according to Equation (1), the transverse magnification M can be increased to reduce the electron beam angle on the image side  β to obtain an electron optical system with high imaging quality, as shown in Figure 1. Furthermore, increasing the transverse magnification M requires the tube length to be increased. However, the long tube length will lead to the large size of the splitter tube, which is not conducive to shortening the overall transit time of the electron beam and will limit its physical temporal resolution. Additionally, the final forming machine will be huge if the time-broadening framing unit is added to the rear end, which is not conducive to being carried on the Shen-guang III experimental platform. Therefore, the lateral magnification can be set to −2 to −3.

The minimum size of the existing MCP traveling wave gate detector is approximately 40 mm [20,21,22,23,24]. Therefore, the minimum diameter of the rear fluorescent screen of the streak tube is required to be 40 mm. The original intention of the design of this tube type was to achieve at least nine subframes (3 × 3). Considering the spacing between images and the limitation of the effective size of the fluorescent screen, it is required that the maximum size of each image should not exceed 12 mm × 12 mm. Furthermore, the effective working area on the object surface must be at least 4 mm × 4 mm in combination with the constraints of the above lateral magnification. Additionally, the effective emission surface of the electronic optical imaging system should work in the paraxial area as much as possible to obtain high-resolution images. In comprehensive consideration, the pipe diameter should be set to 40 mm.

**Figure 2:**
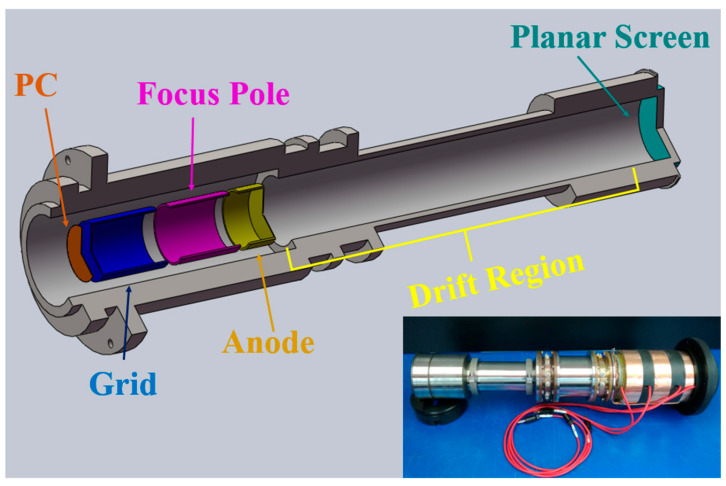
Structural diagram and physical drawing of the streak tube.

The electron optical imaging system was designed according to the aforementioned constraints. The final tube-type structure is shown in Figure 2. The lower right corner is the sample tube. The overall tube length is 460 mm, the tube diameter is 40 mm, the transverse magnification is   M≈−3, the total anode voltage is 10 kV, the length of the focus area is 135 mm, the length of the drift area is 325 mm, and the field strength between the cathode and the grid is 2 kV/mm. The voltage parameters of each electrode are shown in Table 1.

## 3. Development and Experimental Test of the Sample Tube

### 3.1. Preparation of Photocathode

**Figure 3:**
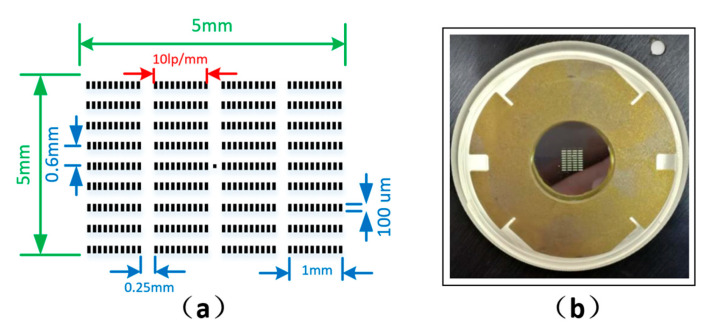
Picture of the pattern and the photocathode: (**a**) metal chromium reticle pattern; (**b**) photocathode assembly.

The metal chromium reticle was first manufactured by outsourcing to facilitate the measurement of the spatial resolution of the streak tube, with an overall dimension of 5 mm × 5 mm. In this range, 9 × 4 slits, with widths of 100 µm and lengths of 1 mm, were processed. Each slit had a corresponding spatial resolution of 10 lp/mm. The specific structural parameters are shown in Figure 3a, where the central dot of the image is the central marking point. Thereafter, we used the reticle to carve the corresponding pattern on the aluminum film substrate and to prepare C_8_H_8_ on the pattern and the support film. Furthermore, we used the electron beam evaporation method to coat the 80 nm thick Au. The prepared X-ray transmission photocathode is shown in Figure 3b.

### 3.2. Development of Fluorescent Screen Components

The common substrate of fluorescent screens is a glass panel or an optical fiber panel. Optical fiber panels are used to increase the light energy coupling between the fluorescent screen and the subsequent image-recording device, the charge-coupled device (CCD), because the streak tube is mostly used for weak light detection to reduce the loss of light energy.

The particle size of the phosphor has a direct relationship with the spatial resolution of the fluorescent screen; the smaller and more uniform the phosphor particles are, the stronger the phosphor is bonded to the substrate. The closer the contact is, the denser the screen’s surface is. The smaller the light scattering is, the higher the spatial resolution of the prepared fluorescent screen is. However, the particle size of phosphor is insignificant and reduces its luminous efficiency, thus affecting the brightness of the screen.

**Figure 4:**
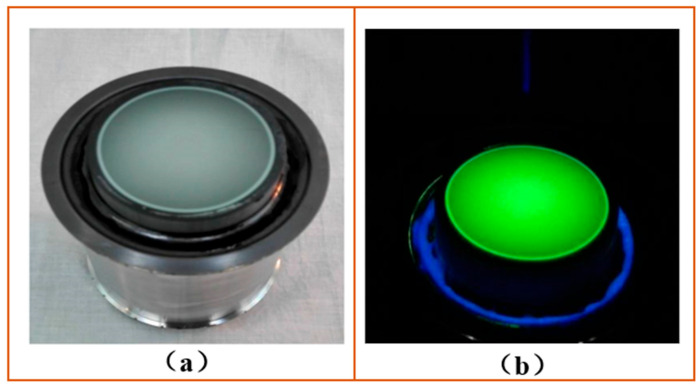
Fluorescent screen assembly and light emission: (**a**) planar fluorescent screen assembly; (**b**) luminescence of the fluorescent screen under ultraviolet lamp irradiation.

The final image of the streak tube is observed by the human eye, which is most sensitive to the spectrum of yellow and green light. Furthermore, the response wavelength of the subsequent CCD is also the best for the yellow–green light spectrum. Therefore, the phosphor selected must be the one that is sensitive to the yellow–green light. In addition, the screen prepared by the selected phosphor must have a high spatial resolution to avoid affecting the spatial resolution of the streak tube. Based on various factors, P20 phosphor with a particle center radius of 5 µm and sensitivity to yellow–green light was finally selected to prepare a planar fluorescent screen. The final prepared fluorescent screen assembly and its luminescence are shown in Figure 4.

## 4. Test Results and Analysis

**Figure 5:**
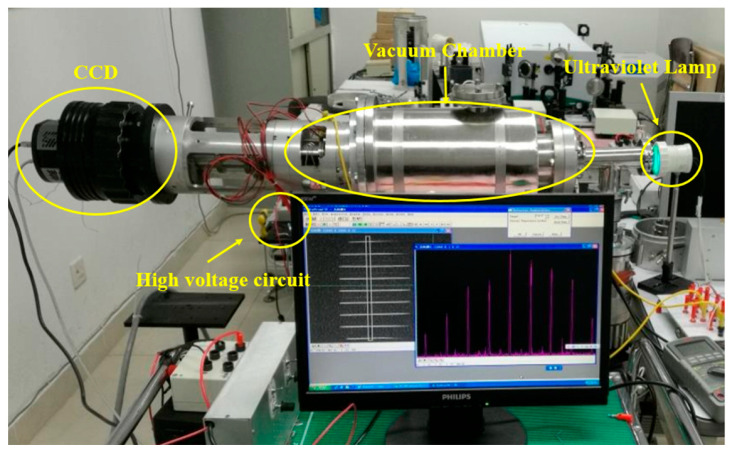
Experimental test platform and streak tube.

Measurement of waist position and beam size. Figure 5 shows the experimental conditions during the experimental test. The sample tube was placed in the vacuum chamber. Furthermore, only its static spatial resolution was required to be measured because the dynamic performance of the reticle only relied on the timing performance of the MCP traveling wave gated reticle unit equipped in the later stage [25,26,27,28]. Therefore, only the ultraviolet lamp was used as the light source during the test.

### 4.1. Measurement of Beam Waist Position

**Figure 6:**
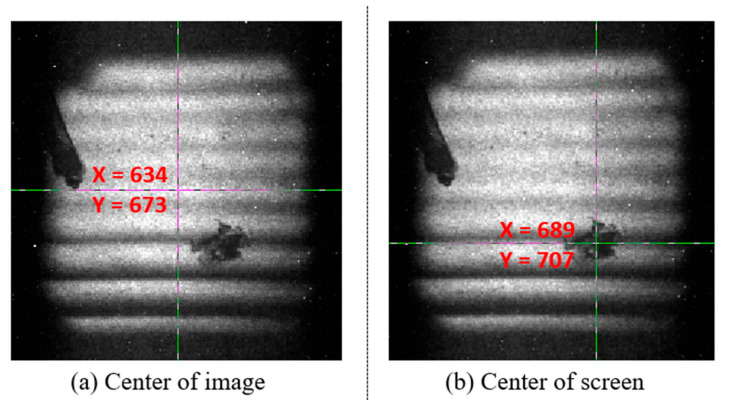
Measurement results of the beam waist offset: (**a**) center of image; (**b**) center of screen. (The values of X and Y in the figure correspond to the pixel coordinates of the position represented by the green cross in the image from the CCD).

As this paper is designed for SLOS technology, the means to realize multi-frame framing in the later stage is also to use the mechanical cutting image dissector structure. If the central position of the electron beam and the image dissector are misaligned with each other in this framing technology, the information of each sub-image obtained by the final framing from our previous tracking experiment will be lost [18]. Therefore, the position of the beam spot needed to be measured. During the test, we made a mark at the center of the used fluorescent screen assembly. Thereafter, we directly connected the fluorescent screen to the rear end of the outlet of the anode diaphragm (the entrance of the drift zone). The image recording system was still CCD (Teledyne Princeton Instruments, 3660 Quakerbridge Road, Trenton, NJ, USA), with the pixel size being (model PI 1340F) 30 µm. The test results are shown in Figure 6.

From the results in Figure 6, the differences between the image center and the center of the fluorescent screen were  Δx=55 pixel×30 µm=1.65 mm  and  Δy=34 pixel×30 µm=1.02 mm. The reasons for the deviation may be analyzed as follows: first, there was the impact of assembly accuracy, and second, there were various magnetic fields in the surrounding environment, such as the geomagnetic field [29]; another explanation could be that the devices (such as screws) used in the assembly process of the stripe tube had weak magnetism.

### 4.2. Beam Spot Position Adjustment and Size Measurement

The elimination of the geomagnetic field was achieved by adding a geomagnetic shielding cavity in the later stage, and the elimination of the effects of small parts with weak magnetism was achieved by replacing the corresponding parts. However, the mold that was used will inevitably lead to errors in the manual assembly processes because the streak tube was assembled manually, despite being fine. 

**Figure 7:**
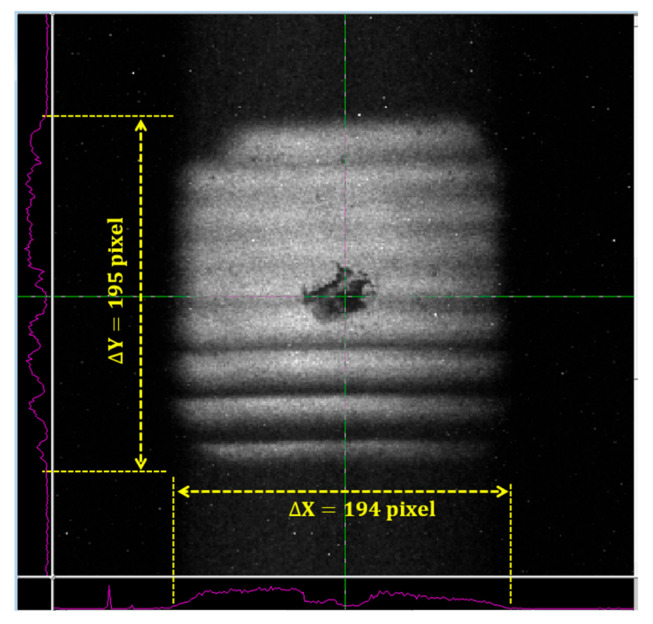
The position and size of the rear waist after adjustment. (The yellow arrow in the figure indicates the number of pixels corresponding to each length.)

Therefore, it was necessary to add a pair of electric displacement plates in the X and Y direction at the rear end of the anode diaphragm to realize the correction of the beam spot position during the implementation of SLOS technology. The position of the beam spot after correction is shown in Figure 7. It can be seen that the image center almost coincides with the center of the fluorescent screen.

In addition, according to the size of the beam spot shown in Figure 7, the number of pixels occupied by the side length of the beam spot was 194 pixels. When multiplied by the pixel size of the CCD, the corresponding size of the beam spot size should be  194 pixel ×30 μm=5.82mm. It can be found that the size of the photocathode pattern included was 5mm × 5mm, from which the size ratio between the waist and the object could be calculated to be 1.16:1, while the ratio of the streak tube used in the early tracking study of SLOS technology was only 0.72:1 [18], with the same photocathode pattern.

### 4.3. Measurement of Static Spatial Resolution

**Figure 8:**
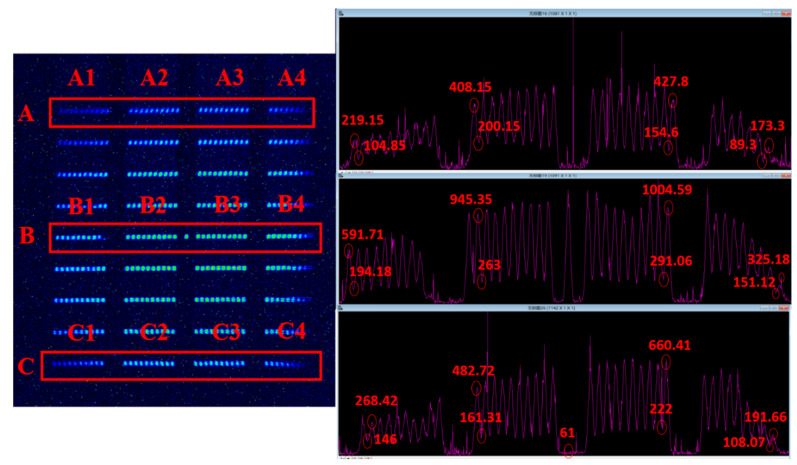
Streak image (left) and intensity curve (right): The purple curves in the right figure are the distributions of the average values of intensity along the horizontal direction of the selected part in the red boxes in the left image, where the abscissa are the pixel positions, and the ordinate is the intensity value. The red numbers represent the strength values of the positions shown in the red circle.

As mentioned above, the consequence of a large beam waist radius is that the intensification of field curvature leads to the deterioration of image quality; therefore, it may still be necessary to measure the spatial resolution of the streak tube designed in this study. Because the CCD model PI 1340F used above was limited by the pixel size, the maximum spatial resolution was only 15 lp/mm. In order to ensure better high-frequency details during this indicator test, the CCD model was replaced with PI 2048F (Teledyne Princeton Instruments, 3660 Quaker bridge Road, Trenton, NJ, USA, pixel size 13.5 µm), and the image obtained by the static experiment is shown in Figure 8. We read the intensity curves of streak images in A, B, and C areas, selected in the frame in the text through the Winview (for 32bit Windows, Version: 2.6.18.0, Microsoft, Redmond, WA, USA) image-reading software of the CCD used to obtain the peak and valley values of their respective intensity curves, and calculated the optical contrast of their corresponding images at this moment through the following formula. The calculated results are shown in Table 2.

(2) $$C=\frac{I_{max}-I_{min}}{I_{max}+I_{min}-2\times I_{nos}}$$

where $I_{max}$, $I_{min}$ and $I_{nos}\;$ refer to the peak value, valley value, and background noise in the intensity curve, respectively. Figure 8 shows the lateral magnification of the streak tube measured from the fringe pattern. According to the data in Figure 3a and Table 2, the static spatial resolution can reach at least 10 lp/mm @ CTF 42% within the effective range of the whole cathode.

**Table 2:** Intensity peaks, valleys and contrasts corresponding to areas A, B, and C in the image.

Block	A				B				C			
Group	A1	A2	A3	A4	B1	B2	B3	B4	C1	C2	C3	C4
$I_{nos}$	61											
$I_{max}$	219.15	408.15	427.8	173.3	591.71	945.35	1004.59	325.18	268.42	482.72	660.41	191.66
$I_{min}$	104.85	200.15	154.6	89.3	194.18	263	291.06	151.12	146	161.31	222	108.07
**C**	0.566	0.428	0.593	0.597	0.599	0.628	0.608	0.491	0.419	0.616	0.577	0.470

### 4.4. Measurement of Transverse Magnification

**Figure 9:**
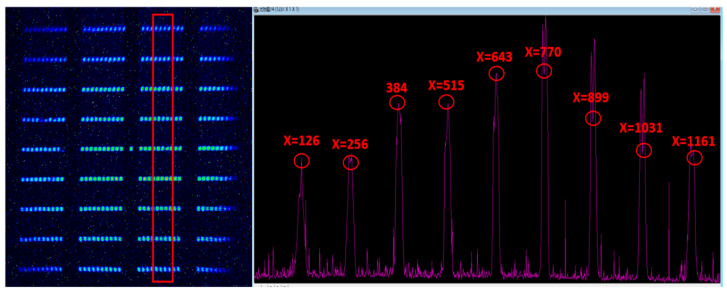
Measurement of transverse magnification: The purple curve in the right figure is the distribution of the average value of intensity along the horizontal direction of the selected part in the red box in the left image, where the abscissa are the pixel positions, and the ordinate is the intensity value. The red numbers represent the pixel coordinates of the positions shown in the red circles.

Figure 9 shows the values taken along the longitudinal direction in the experimental diagram. We checked the peak coordinates (the part on the right side of Figure 7). The spacing between the streak images could be obtained according to the difference between the coordinates and the product of the pixel size (13.5 µm) of the CCD used. The spacing range was [1.728, 1.755] mm, with an average of 1.742 mm. Combined with Figure 2a, the physical spacing between the original streak pattern was 0.6 mm. Therefore, the transverse magnification could be $\;\;\;M=\frac{1.742\mathrm{mm}}{0.6\;\mathrm{mm}}\approx 2.9\;$.

Table 3 shows the comparison of the parameters of the large-waist striped tube designed in this paper and the streak tube used in the follow-up study of SLOS technology in the early stage. The SLOS technology based on “mechanical cutting” could only achieve the four-frame result, due to the limitation of the waist size. In the early tracking research, the waist-to-object size ratio corresponding to this result was 0.72:1. However, the design in this paper not only achieved the waist-to-object size ratio of 1.16:1, it also retained the high-resolution ability. We believe that it can promote the further development of SLOS framing technology into multi-frame imaging.

## 5. Conclusions

SLOS technology is limited by its image dissector and can only achieve up to four frames of experimental results. This study obtained the design idea of a large-waist streak tube by increasing the lateral magnification of the streak tube and increasing the tube length advance based on the analysis of the Lagrange–Helmholtz relationship to improve the number of framing frames of SLOS technology to realize multi-framing in situ. Furthermore, it designed and developed a sample tube for experimentation on this basis. The test results showed that, under the assumption of having a large-waist electron beam radius, the static spatial resolution of the tube type could reach 10 lp/mm (@ 42%) on average. Moreover, the lateral magnification could reach −2.9. Compared with the previous tracking research results, if the MCP traveling wave gating unit is equipped at the output end, it is expected to promote the further development of SLOS technology.

The size of the photocathode used in this test was only 5mm × 5mm, compared with its pipe diameter (40 mm). The electron beam emitted by the entire photocathode reticle was actually working in the paraxial region, which was also the reason for its high spatial resolution. However, in contrast, one could find that the effect of the large waist-to-object size ratio may limit the actual effective detection area of the photocathode, which was also the deficiency of this study. Therefore, one of our follow-up research projects should be to coordinate the detection area of the streak tube with the realization of the SLOS multi-frame technology.

## Figures and Tables

**Table 1:** Voltage of the poles.

Pole	PC	Grid	Focus Pole	Anode
Voltage (kV)	−10	−6	−8.24	0

**Table 3:** Comparison of the imaging performance of the streak tube.

Parameters	Streak Tube in This Study	Streak Tube in Literature
Tube Length (mm)	460	424
Diameter of Tube (mm)	40	60
Static Spatial Resolution (lp/mm)	10 (@CTF 42%)	15 (@CTF 20%)
Waist-to-object size ratio	1.16:1	0.72:1
Transverse magnification	2.9	1.32

## Data Availability

The data underlying the results presented in this study are not currently publicly available but may be obtained from the authors upon request.
